# Association Between Neutrophil Percentage-to-Albumin Ratio and Depression in Middle-Aged and Elderly Adults: A National Study

**DOI:** 10.1155/bn/4199054

**Published:** 2025-03-27

**Authors:** Leiyong Zhao, Chengjun Li, Hequn Lv, Chunli Zeng, Yongjun Peng

**Affiliations:** ^1^Department of Acupuncture and Rehabilitation, Affiliated Hospital of Nanjing University of Chinese Medicine, Nanjing, Jiangsu Province, China; ^2^Department of Neurology, Huangdao District Hospital of Traditional Chinese Medicine, Qingdao, Shandong Province, China; ^3^Department of Acupuncture, Yancheng TCM Hospital Affiliated to Nanjing University of Chinese Medicine, Yancheng, Jiangsu Province, China; ^4^Department of Lung Disease, Yancheng TCM Hospital Affiliated to Nanjing University of Chinese Medicine, Yancheng, Jiangsu Province, China

**Keywords:** association, depression, large-scale, national study, NPAR, U-shaped

## Abstract

**Background:** The association between inflammatory markers and depression has garnered increasing attention. The neutrophil percentage-to-albumin ratio (NPAR) is an emerging inflammatory marker, but its association with depression in middle-aged and elderly adults was not previously explored. The purpose of this study was to investigate the association through a national study in the United States.

**Methods:** All study data were weighted to ensure representativeness. Multivariate logistic regression models were applied to explore the independent relationship of NPAR with depression in middle-aged and elderly adults. Restricted cubic splines were employed to examine potential nonlinear association, with turning points calculated using a recursive algorithm upon detecting nonlinearity. Stratified analyses and interaction tests were conducted to explore subgroup differences.

**Results:** In the model adjusted for all confounding factors, no significant relationship was found between NPAR and depression in middle-aged and elderly adults [1.02 (0.92, 1.12)]. Further sensitivity analysis indicated a potential U-shaped relationship between NPAR and depression in middle-aged and elderly adults, with the OR (95% CI) of 0.74 (0.60, 0.92), 0.87 (0.70, 1.08), 0.92 (0.72, 1.19) for Q2, Q3, and Q4, respectively, compared to Q1. The U-shaped association was confirmed by the restricted cubic spline. Subsequent analysis identified an inflection point at 14.05, revealing inverse relationships before and after this point. Subgroup analysis indicated sex differences in this association.

**Conclusion:** This large-scale cross-sectional study identified a U-shaped association between NPAR and depression in American middle-aged and elderly adults.

## 1. Introduction

Depression, identified as a leading mental health disorder globally, significantly affects the quality of life and daily functioning of those afflicted [1, 2]. Its profound impact extends beyond psychological health, exhibiting strong associations with several physical conditions, including cardiovascular diseases (CVD), metabolic syndrome, and disturbances in immune function [3, 4]. It is estimated that depression affects approximately 264 million individuals worldwide, with its prevalence demonstrating a rising trend [5]. Despite extensive investigations into its etiology and pathophysiology, the precise biological mechanisms underlying depression remain to be fully delineated. Recent evidence increasingly supports a significant role of inflammatory processes in the pathogenesis of depression. Studies indicate that individuals with depression frequently present with low-grade chronic inflammation, underscoring inflammation as a pivotal factor in both the development and progression of the disorder [6, 7]. Moreover, the prevalence of depression is significantly higher in patients with immunoinflammatory-related diseases than in the normal population [8, 9]. An animal experiment confirmed that proinflammatory factors could increase the permeability of the blood–brain barrier and promote the infiltration of peripheral inflammatory factors and immune cells, which exacerbated depressive-like behaviors [10]. Those growing understandings could potentially inform novel therapeutic strategies targeting inflammatory pathways in depression management.

Neutrophil percentage and serum albumin have recently attracted considerable attention in psychiatric and immunological research. Neutrophils, predominant among white blood cells (WBCs) within the immune system, play a crucial role in inflammatory responses [11]. Elevated neutrophil levels, typically observed in various chronic inflammatory conditions and acute stress reactions, have been positively correlated with depression severity, suggesting neutrophils' significance as a biomarker for depression [12]. A study revealed that mental stress could lead to an increase in cerebral neutrophils and upregulation of the neutrophil-specific surface molecule CD177 expression in peripheral neutrophils in mice, leading to endothelial disruption of the cerebral vasculature and causing depressive manifestations [13]. Albumin, a plasma protein synthesized in the liver and a standard marker for assessing nutritional and overall health status, is linked to systemic inflammation and immune dysfunction when at low levels [14]. Studies have demonstrated that albumin levels were significantly lower in patients with depression compared to healthy controls, underscoring its potential role in this condition [15, 16].

Although research on the roles of neutrophil percentage and serum albumin in depression is expanding, existing studies predominantly focus on isolated biomarkers and lack a multidimensional integrated analysis. The neutrophil percentage-to-albumin ratio (NPAR) has emerged as a biomarker of increasing interest in medical research, indicating inflammatory status to potentially elucidate disease risk and prognosis [17]. Currently, no studies explicitly explore the association between NPAR and depression in middle-aged and elderly adults. Consequently, this research is aimed at addressing this gap by conducting a large-scale cross-sectional analysis to systematically evaluate the association between NPAR and depression in middle-aged and elderly adults.

## 2. Methods

### 2.1. Study Population

**Figure 1:**
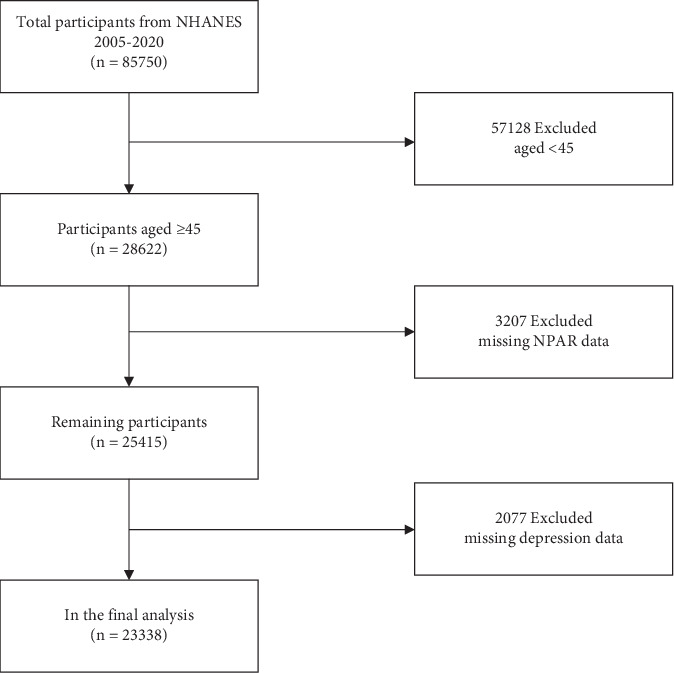
Flow chart of sample selection.

The National Health and Nutrition Examination Survey (NHANES) is a critical initiative led by the National Center for Health Statistics (NCHS) under the auspices of the Centers for Disease Control and Prevention (CDC). NHANES is instrumental in delineating the health and nutritional status of the American population. It integrates structured interviews and detailed physical examinations to gather extensive health-related data, establishing it as an invaluable resource for public health research and policy development. NHANES utilizes a sophisticated multistage probability sampling technique to select a representative sample across the US demographic spectrum. Furthermore, NHANES data are publicly accessible via the website (https://wwwn.cdc.gov/nchs/nhanes), providing a vital resource for researchers and policymakers to conduct secondary analyses that enhance the breadth of epidemiological research. In this study, exclusions were applied to individuals under 45 years of age (*n* = 57,128), those with missing NPAR data (*n* = 3207), and those lacking depression data (*n* = 2077). Ultimately, 23,338 participants were included in the analysis (Figure 1).

### 2.2. Assessment of NPAR

The neutrophil percentage and serum albumin were derived from blood test data. All blood tests were conducted on participants in a fasting state. NPAR was calculated by dividing the neutrophil percentage by the serum albumin level [18, 19], using the following formula: NPAR = Neutrophil Percentage (%)∗100/Albumin (g/dL).

### 2.3. Assessment of Depression

In the NHANES database, the assessment of depression was conducted using the standardized Patient Health Questionnaire-9 (PHQ-9), which was extensively used in public health research to identify the prevalence of depression, associated risk factors, and its correlations with other health conditions [20, 21]. The PHQ-9 included nine questions, each based on the diagnostic criteria for depression from the Diagnostic and Statistical Manual of Mental Disorders. Scores for each question ranged from 0 to 3, reflecting the frequency of symptoms over the past 2 weeks, with a total possible score ranging from 0 to 27. Individuals who scored a 10 or more were assessed to be suffering from depression. This definition had 88% sensitivity and 88% specificity, indicating a high degree of accuracy [20, 22].

### 2.4. Assessment of Covariates

To mitigate the impact of confounding factors on the results, we included a comprehensive range of covariates. Demographic factors incorporated were sex, age, race, marital status, education, and income-to-poverty ratio. Lifestyle variables included drinking status, smoking status, and vigorous recreational activities. To control for the interference of chronic diseases, we included conditions such as stroke, chronic kidney disease (CKD), CVD, hyperlipidemia, diabetes, and hypertension. The use of antidepressant and anti-inflammatory drugs was also enrolled for further adjustment. Body measurements included body mass index (BMI) and waist circumference. Blood markers related to inflammation, such as WBCs, lymphocytes, monocytes, neutrophils, uric acid, and C-reactive protein (CRP), were also adjusted for in the analysis.

### 2.5. Statistical Analysis

Due to the complex sampling methodology employed in the NHANES, we conducted a weighted analysis to ensure the representativeness of our findings for the U.S. population. All statistical analyses were carried out using R software (Version 4.3.2) and EmpowerStats. Hypothesis testing was performed using two-sided tests, with a significance threshold set at *p* < 0.05. We categorized the study population into four groups based on the quartiles of NPAR for descriptive analysis at baseline. Continuous variables were expressed as medians (standard errors) and analyzed via weighted linear regression. Categorical variables were reported as percentages (standard errors), and assessed using weighted chi-square tests.

Depression was analyzed as a dichotomous outcome. Logistic regression models were employed to explore the relationship between NPAR and depression in middle-aged and elderly adults, with adjustments made for multiple confounders to mitigate potential biases. We performed sensitivity analyses using quartile-based stratification of NPAR to examine the robustness of our findings. To explore potential nonlinear relationships, we applied restricted cubic spline analysis. Upon detecting nonlinearity, a recursive algorithm was utilized to determine the inflection point, followed by the construction of a piecewise regression model to examine the association between NPAR and depression both before and after this point. We also conducted stratified analyses and interaction tests to identify any differences among specific subgroups.

## 3. Results

### 3.1. Baseline Characteristics

In this research, the participants were divided into four groups based on NPAR, as presented in Table 1. The average age of the population was 60.51(0.14) years, with 52.40% being women. White and Black individuals comprised 74.40% and 9.44% of the study population, respectively. When compared to other groups, individuals in Q4 were more likely to be female, older, individuals using antidepressants, and current smokers and exhibited higher BMI and waist measurements, coupled with a lower income-to-poverty ratio. These individuals showed elevated levels of WBC, neutrophils, monocyte, neutrophil percent, and CRP. They were generally at a greater risk for CKD, CVD, stroke, hypertension, diabetes, and depression.

### 3.2. Association Between NPAR and Depression

**Figure 2:**
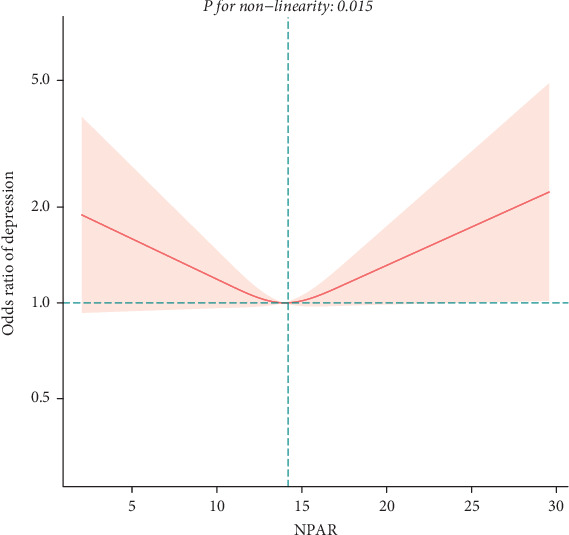
Association between NPAR and depression in middle-aged and elderly adults. Age, sex, race, education level, income-to-poverty ratio, marital status, smoking status, drinking status, vigorous recreational activity, BMI, waist, stroke, CKD, CVD, hyperlipidemia, diabetes, hypertension, WBC, lymphocyte, monocyte, neutrophils, uric acid, and CRP were adjusted.

To exclude the influence of confounding factors, we developed three logistic regression models (Table 2). In the unadjusted model (Model 1) and the model adjusted only for age, sex, and race (Model 2), NPAR was positively correlated with depression in middle-aged and elderly adults, with odds ratios (OR) and 95% confidence intervals (CI) of 1.20 (1.15, 1.26) and 1.24 (1.18, 1.29), respectively. In Model 3 adjusting for all covariates, we found no significant association between NPAR and depression [1.02 (0.92, 1.12)]. Subsequent sensitivity analysis revealed a potential U-shaped relationship between NPAR and depression in middle-aged and elderly adults, as indicated by OR (95% CI) of 0.74 (0.60, 0.92), 0.87 (0.70, 1.08), 0.92 (0.72, 1.19) for Q2, Q3, and Q4, respectively, compared to Q1. We confirmed the presence of a U-shaped relationship using the restricted cubic spline (Figure 2) and identified an inflection point at 14.05 (Table 3), showing opposite relationships before and after this point. Before the inflection point, NPAR was negatively correlated with depression [0.84 (0.70, 0.99)]; after the inflection point, a positive correlation was observed [1.13 (0.99, 1.28)].

### 3.3. Subgroup Analyses

**Figure 3:**
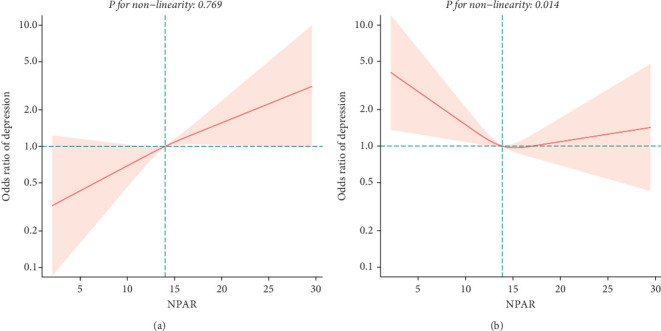
Association between NPAR and depression in middle-aged and elderly adults by sex ((a) men; (b) women).

**Figure 4:**
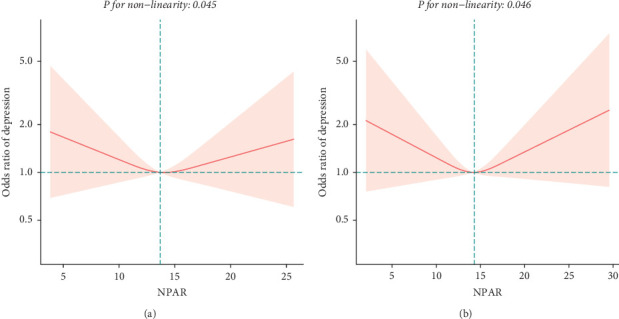
Association between NPAR and depression stratified in middle-aged and elderly adults by age ((a) < 60; (b) ≥ 60).

To explore potential differences in the association between the NPAR and depression across various subgroups, we conducted stratified analyses and interaction tests. In sex-stratified analysis, we found a positive correlation between NPAR and depression in middle-aged and elderly men [1.24 (1.06, 1.46)] and no significant correlation in middle-aged and elderly women [0.91 (0.77, 1.09)] (Table 2). Further analysis using restricted cubic splines showed that the relationship between NPAR and depression in men was linear, whereas it was nonlinear in women (Figure 3). In age-stratified analysis, the relationship between NPAR and depression was not statistically significant for individuals aged < 60 years old or those aged ≥ 60 years of age (Table 2). The stratified restricted cubic splines indicated that the relationship in both age subgroups was consistent with the overall U-shaped trend (Figure 4). In subgroups based on race, lifestyle factors, and chronic diseases, no significant interactions were detected (Table 4).

## 4. Discussion

In this large-scale study, we made the investigation of the association between NPAR and depression among the American middle-aged and elderly population. The findings revealed a U-shaped relationship between NPAR and depression in the general middle-aged and elderly adults, with an inflection point at 14.05. Before and after the point, the association between NPAR and depression was statistically significant, exhibiting inverse relationships. Stratified analysis by sex revealed differences in the relationship between NPAR and depression among men and women, while age-stratified analysis indicated that the relationship was nonlinear in these subgroups. Additionally, interaction tests did not reveal any significant interactions. This study may provide novel insights into the complex interactions between inflammation and mental health in a diverse population.

This study identified a U-shaped association between NPAR and depression in the middle-aged and elderly population, which implied that an increase in NPAR was associated with a decrease in depressive symptoms when NPAR was lower than 14.05. In comparison, an increase in NPAR was associated with an increase in depressive symptoms when NPAR was higher than 14.05. The inflection point of 14.05 may serve as an important reference value for identifying individuals at higher risk of depression in the middle-aged and elderly population, which provides clinicians with a potential early warning indicator for timely detection and intervention of depressive symptoms. In public health management, this finding may help optimize resource allocation, and by identifying high-risk populations, healthcare resources could be allocated more efficiently to improve the efficiency of health management.

To our knowledge, this study was the first to explore the relationship between the emerging inflammatory marker, NPAR, and depression in middle-aged and elderly adults. Previous research has predominantly focused on neutrophil count or serum albumin individually. A study from China involving ischemic stroke patients suggested that neutrophil count might be a significant predictor of 1-year poststroke depressive symptoms and was closely related to stroke severity [23]. Similarly, a cross-sectional study in America found a significantly positive correlation between neutrophil count and depression [24]. Research from the United Kingdom indicated an association between depressive symptoms and impaired neutrophil function following hip fractures [25]. A prospective cohort study in Whites showed a significant association between elevated neutrophil counts and depression [26]. In terms of the relationship between serum albumin and depression, studies on elderly stroke survivors showed that low serum albumin levels appeared to be associated with poststroke depressive symptoms [27]. Research from Jordan and America has indicated a negative correlation between serum albumin and depression [28, 29]. In patients with chronic liver disease, lower serum albumin levels are positively correlated with depressive symptoms [30]. Notably, similar conclusions have been drawn from studies involving patients with schizophrenia, HIV infection, and metastatic cancer [31–33]. In a recent study of NPAR and depression in the general adult population, a similar nonlinear relationship was found; however, limited nonlinear subgroup analyses restricted further exploration of gender differences [34]. Furthermore, in a study with a similar index to NPAR, the neutrophil–albumin ratio (NAR), there was a significant association between higher NAR levels and more severe depression in patients with irritable bowel syndrome, which was inconsistent with our findings [35].

In this study, we found a U-shaped association between NPAR and depression in middle-aged and elderly adults, indicating that both extremely low and high levels of NPAR were associated with an increased risk of depression. This complex relationship may involve multiple biological mechanisms and systemic interactions. The first is inflammation and immune responses. Neutrophils are a crucial component of the immune system. Low neutrophil levels may indicate weakened immune function, leading to an increased risk of infections and chronic inflammation, which are closely linked to depression [36]. Moreover, high neutrophil levels are often associated with acute and chronic inflammatory responses [37]. Elevated levels of inflammatory cytokines can damage synapses, reduce synaptic density and function, and impair brain neuroplasticity, thereby increasing the risk of depression [38] Albumin possesses anti-inflammatory and immunomodulatory properties [39]. Reduced albumin levels are typically associated with chronic inflammation and malnutrition, which can exacerbate inflammatory responses and increase the risk of depression [40]. The second is oxidative stress. Neutrophils are one of the primary producers of reactive oxygen species (ROS), which play a pivotal role in inflammatory responses [41]. High neutrophil levels can increase ROS production, leading to oxidative stress [42]. This stress can damage cells, including neurons, thus impairing brain function and triggering depressive symptoms. Albumin, being a major plasma protein with antioxidant properties, can neutralize ROS. Higher albumin levels enhance the body's antioxidant defenses, reducing oxidative stress and protecting the brain from damage [43]. Low albumin levels, on the other hand, diminish antioxidant capacity, further increasing oxidative stress. Third, neurotransmitter metabolism is implicated. Depression is closely related to the imbalance of neurotransmitters such as serotonin (5-HT) [44]. Inflammatory cytokines can affect the metabolic pathways of 5-HT, lowering its levels in the brain and thereby inducing depressive symptoms [45]. Increased neutrophils and inflammatory cytokines exacerbate this effect. Tryptophan, a precursor of 5-HT, can be metabolized through the indoleamine 2,3-dioxygenase (IDO) pathway under the influence of inflammatory cytokines, converting it into kynurenine and reducing the availability of tryptophan for 5-HT synthesis [46]. This reduction in 5-HT levels contributes to increased depressive symptoms [47]. In summary, the relationship between NPAR and depression may involve a multifaceted interplay of mechanisms that interact complexly to influence the onset and progression of depression. Further studies are needed to combine clinical data and basic experiments to validate these hypotheses and explore specific molecular mechanisms.

The sex-stratified analysis indicated men had a higher risk of depression with elevated NPAR levels. This disparity may be related to differences in hormone levels and behavioral patterns. Testosterone, a primary male hormone, has anti-inflammatory properties [48]. Higher levels of testosterone are associated with a relatively weaker immune response and lower inflammation. Consequently, a high NPAR might indicate low testosterone levels, which are linked to chronic inflammation. Chronic inflammation, in turn, is closely associated with various mental health issues, including depression [49]. Moreover, studies suggest that men are more likely to internalize their emotions in response to stress, meaning they are more prone to suppressing negative emotions rather than expressing them or seeking help [50, 51]. This internalization can lead to the accumulation of emotional distress, which may eventually manifest as depressive symptoms [52]. In contrast, women are generally more inclined to express their emotions and seek social support, which can effectively alleviate stress. Traditional gender role expectations might also discourage men from acknowledging mental health problems and seeking professional help. Additionally, men often face greater workplace stress, especially when fulfilling the role of the primary breadwinner for their families. This high level of stress and expectation can lead to chronic stress accumulation, which in turn may trigger chronic inflammation and depressive symptoms [53].

Despite the valuable epidemiological evidence provided by this study, several limitations need to be acknowledged. First, cross-sectional studies can only reveal correlations between variables and cannot establish causality. This design did not allow us to determine whether depressive symptoms are caused by NPAR. Second, the measurement of depressive symptoms in NHANES primarily relies on self-reported questionnaires, which may be subject to reporting bias. Participants might underestimate or overestimate their depressive symptoms due to recall bias or social desirability bias. Third, although this study has thoroughly considered confounding factors, some confounders might still have been omitted due to the limitations of the database. Fourth, while NHANES data are broadly representative, the randomness and sampling design of data collection may result in insufficient representation of certain specific populations. Furthermore, the applicability of these findings to other countries requires further investigation.

## 5. Conclusion

This large-scale study revealed a U-shaped relationship between NPAR and depression in the general American middle-aged and elderly adults. Further stratified analysis uncovered sex differences in this association. This study may potentially provide a reference for alleviating depression by intervening with inflammatory indicators. Future cohort studies and randomized controlled trials are needed to validate these results.

## Figures and Tables

**Table 1:** Baseline characteristics.

**Variable**	**Total (** **n** = 23,338**)**	**Q1 (** **n** = 5841**)**	**Q2 (** **n** = 5815**)**	**Q3 (** **n** = 5847**)**	**Q4 (** **n** = 5835**)**	**p** ** value**
Age (years)	60.51 (0.14)	59.05 (0.20)	59.49 (0.19)	61.03 (0.21)	62.45 (0.24)	< 0.0001
Sex (%)						0.12
Male	47.60 (0.01)	48.65 (0.90)	48.40 (0.79)	47.65 (0.97)	45.70 (0.90)	
Female	52.40 (0.01)	51.35 (0.90)	51.60 (0.79)	52.35 (0.97)	54.30 (0.90)	
Race/ethnicity (%)						< 0.0001
Mexican American	5.51 (0.00)	5.04 (0.49)	6.05 (0.55)	5.46 (0.54)	5.43 (0.50)	
White	74.40 (0.03)	66.50 (1.45)	74.53 (1.22)	77.67 (1.06)	78.29 (1.06)	
Black	9.44 (0.00)	15.91 (0.98)	7.97 (0.55)	6.97 (0.47)	7.47 (0.50)	
Other	10.66 (0.00)	12.56 (0.69)	11.45 (0.66)	9.90 (0.58)	8.80 (0.59)	
Education level (%)						< 0.001
Less than high school	5.64 (0.00)	5.84 (0.43)	5.56 (0.35)	5.62 (0.38)	5.55 (0.35)	
High school	25.65 (0.01)	24.46 (1.00)	24.13 (0.93)	25.43 (0.89)	28.63 (0.90)	
More than high school	68.71 (0.02)	69.70 (1.12)	70.31 (1.02)	68.95 (0.98)	65.82 (0.98)	
Marital status (%)						< 0.0001
Married/living with partner	67.18 (0.02)	69.25 (0.94)	68.05 (1.01)	68.44 (0.87)	62.97 (0.91)	
Divorced/separated/widowed	26.65 (0.01)	23.89 (0.87)	26.09 (0.86)	26.02 (0.84)	30.56 (0.79)	
Never married	6.16 (0.00)	6.86 (0.54)	5.86 (0.41)	5.54 (0.38)	6.47 (0.43)	
Smoking status (%)						< 0.0001
Never	51.61 (0.01)	53.73 (0.97)	53.62 (0.87)	51.03 (0.95)	48.07 (1.15)	
Former	32.07 (0.01)	31.53 (0.94)	31.22 (0.80)	32.06 (0.72)	33.50 (0.98)	
Current	16.32 (0.01)	14.74 (0.58)	15.16 (0.65)	16.91 (0.75)	18.43 (0.81)	
Drinking status (%)						< 0.0001
Never	11.25 (0.00)	11.50 (0.62)	11.25 (0.55)	10.60 (0.58)	11.70 (0.54)	
Former	14.76 (0.01)	13.52 (0.62)	13.63 (0.66)	15.11 (0.66)	16.76 (0.80)	
Current	73.99 (0.02)	74.98 (0.91)	75.12 (0.95)	74.30 (0.85)	71.53 (1.02)	
Vigorous recreational activities (%)						< 0.0001
Yes	52.11 (0.01)	45.73 (1.13)	49.23 (1.14)	52.26 (1.03)	61.06 (1.04)	
No	47.89 (0.01)	54.27 (1.13)	50.77 (1.14)	47.74 (1.03)	38.94 (1.04)	
Hyperlipidemia (%)						0.004
Yes	18.59 (0.01)	17.85 (0.69)	17.45 (0.79)	18.12 (0.78)	21.01 (0.83)	
No	81.41 (0.02)	82.15 (0.69)	82.55 (0.79)	81.88 (0.78)	78.99 (0.83)	
CKD (%)						< 0.0001
Yes	21.38 (0.01)	14.91 (0.63)	17.84 (0.79)	22.42 (0.82)	30.18 (0.72)	
No	78.62 (0.02)	85.09 (0.63)	82.16 (0.79)	77.58 (0.82)	69.82 (0.72)	
CVD (%)						< 0.0001
Yes	14.76 (0.00)	11.16 (0.58)	11.80 (0.54)	15.03 (0.65)	21.03 (0.77)	
No	85.24 (0.02)	88.84 (0.58)	88.20 (0.54)	84.97 (0.65)	78.97 (0.77)	
Stroke (%)						< 0.0001
Yes	5.00 (0.00)	3.35 (0.26)	3.80 (0.29)	5.34 (0.33)	7.47 (0.47)	
No	95.00 (0.02)	96.65 (0.26)	96.20 (0.29)	94.66 (0.33)	92.53 (0.47)	
Diabetes (%)						< 0.0001
Yes	32.52 (0.01)	27.66 (0.87)	28.48 (0.77)	33.80 (0.90)	40.08 (0.93)	
No	67.48 (0.02)	72.34 (0.87)	71.52 (0.77)	66.20 (0.90)	59.92 (0.93)	
Hypertension (%)						< 0.0001
Yes	53.63 (0.01)	49.76 (1.05)	51.75 (0.95)	53.39 (1.27)	59.54 (0.97)	
No	46.37 (0.01)	50.24 (1.05)	48.25 (0.95)	46.61 (1.27)	40.46 (0.97)	
Depression (%)						< 0.0001
Yes	7.76 (0.00)	6.59 (0.46)	6.61 (0.48)	7.54 (0.49)	10.33 (0.50)	
No	92.24 (0.02)	93.41 (0.46)	93.39 (0.48)	92.46 (0.49)	89.67 (0.50)	
Anti-depressant drug use (%)						< 0.0001
Yes	5.83 (0.00)	3.97 (0.44)	4.97 (0.41)	5.74 (0.56)	8.31 (0.66)	
No	94.17 (0.02)	96.03 (0.44)	95.03 (0.41)	94.26 (0.56)	91.69 (0.66)	
Anti-inflammatory drug use (%)						0.43
Yes	6.86 (0.00)	6.93 (0.46)	6.81 (0.55)	6.29 (0.53)	7.45 (0.44)	
No	93.14 (0.02)	93.07 (0.46)	93.19 (0.55)	93.71 (0.53)	92.55 (0.44)	
BMI (kg/m^2^)	29.54 (0.08)	28.37 (0.11)	29.14 (0.12)	29.75 (0.14)	30.84 (0.15)	< 0.0001
Waist (cm)	102.30 (0.20)	99.02 (0.28)	101.24 (0.30)	103.16 (0.31)	105.62 (0.34)	< 0.0001
Income-to-poverty ratio	3.24 (0.03)	3.30 (0.04)	3.33 (0.04)	3.27 (0.04)	3.07 (0.04)	< 0.0001
WBC (1000 cells/uL)	7.15 (0.03)	6.44 (0.06)	6.80 (0.03)	7.23 (0.04)	8.09 (0.05)	< 0.0001
Lymphocyte (1000 cells/uL)	2.06 (0.01)	2.58 (0.05)	2.12 (0.01)	1.91 (0.01)	1.67 (0.01)	< 0.0001
Monocyte (1000 cells/uL)	0.57 (0.00)	0.57 (0.00)	0.57 (0.00)	0.57 (0.00)	0.58 (0.00)	0.001
Neutrophils (1000 cells/ul)	4.26 (0.02)	3.03 (0.02)	3.86 (0.02)	4.50 (0.03)	5.60 (0.04)	< 0.0001
Uric acid (mg/dL)	5.52 (0.02)	5.49 (0.03)	5.52 (0.02)	5.50 (0.03)	5.56 (0.03)	0.28
Neutrophils percent	58.87 (0.11)	47.56 (0.11)	56.57 (0.09)	61.99 (0.11)	68.78 (0.11)	< 0.0001
Serum albumin (g/dL)	4.19 (0.01)	4.34 (0.01)	4.28 (0.01)	4.18 (0.01)	3.96 (0.01)	< 0.0001
CRP (mg/dL)	2.26 (0.06)	1.31 (0.05)	1.58 (0.06)	2.02 (0.07)	4.15 (0.20)	< 0.0001

Abbreviations: BMI, body mass index; CKD, chronic kidney disease; CRP, c-reactive protein; CVD, cardiovascular disease; WBC, white blood cell.

**Table 2:** Association between NPAR and depression in middle-aged and elderly adults.

**Exposure**	**Model 1 OR (95% CI)**	**Model 2 OR (95% CI)**	**Model 3 OR (95% CI)**
NPAR. *Z* score	1.20 (1.15, 1.26)	1.24 (1.18, 1.29)	1.02 (0.92, 1.12)
Q1	1.0	1.0	1.0
Q2	0.94 (0.82, 1.08)	0.94 (0.82, 1.08)	0.74 (0.60, 0.92)
Q3	1.10 (0.96, 1.25)	1.14 (1.00, 1.30)	0.87 (0.70, 1.08)
Q4	1.54 (1.37, 1.75)	1.65 (1.46, 1.87)	0.92 (0.72, 1.19)
P for trend	< 0.0001	< 0.0001	0.8966
Sex			
Men	1.26 (1.18, 1.35)	1.31 (1.22, 1.41)	1.24 (1.06, 1.46)
Women	1.16 (1.10, 1.23)	1.19 (1.12, 1.26)	0.91 (0.77, 1.09)
Age			
< 60	1.32 (1.23, 1.41)	1.29 (1.21, 1.38)	0.99 (0.82, 1.20)
≥ 60	1.15 (1.08, 1.22)	1.22 (1.15, 1.30)	1.02 (0.88, 1.18)

*Note:* Model 1: No covariates were adjusted. Model 2: .Age, sex, and race were adjusted; Model 3: age, sex, race, education level, income-to-poverty ratio, marital status, smoking status, drinking status, vigorous recreational activity, BMI, waist, stroke, CKD, CVD, hyperlipidemia, diabetes, hypertension, antidepressant drug use, anti-inflammatory drug use, WBC, lymphocyte, monocyte, neutrophils, uric acid, and CRP were adjusted. In the subgroup analysis stratified by age or sex, the model is not adjusted for the stratification variable itself.

**Table 3:** Threshold effect analysis of NPAR on depression in middle-aged and elderly adults using a two-piecewise linear regression model.

	**Adjust OR (95% CI)**	**p** ** value**
Fitting by a standard linear model	1.02 (0.92, 1.12)	0.72
Fitting by two-piecewise linear model		
Inflection point	14.05	
< 14.05	0.84 (0.70, 0.99)	0.03
> 14.05	1.13 (0.99, 1.28)	0.04
Log-likelihood ratio	0.011	

*Note:* Age, sex, race, education level, income-to-poverty ratio, marital status, smoking status, drinking status, vigorous recreational activity, BMI, waist, stroke, CKD, CVD, hyperlipidemia, diabetes, hypertension, antidepressant drug use, anti-inflammatory drug use, WBC, lymphocyte, monocyte, neutrophils, uric acid, and CRP were adjusted.

**Table 4:** Subgroup analysis of risk factors for the relationship between NPAR and depression in middle-aged and elderly adults.

**Variables**	**OR (95% CI)**	**p**	**P for interaction**
Race/ethnicity			0.66
Mexican American	1.24 (0.88, 1.75)	0.22	
White	1.10 (0.92, 1.32)	0.28	
Black	0.96 (0.72, 1.30)	0.81	
Other	0.98 (0.71, 1.34)	0.89	
Smoking status			0.09
Current	0.97 (0.83, 1.14)	0.73	
Former	1.27 (0.99, 1.62)	0.06	
Never	0.89 (0.72, 1.10)	0.28	
Drinking status			0.95
Current	0.97 (0.66, 1.45)	0.89	
Former	0.99 (0.77, 1.27)	0.93	
Never	1.02 (0.91, 1.15)	0.69	
Vigorous recreational activities			0.08
Yes	0.98 (0.88, 1.09)	0.69	
No	1.18 (0.97, 1.44)	0.09	
Hyperlipidemia			0.26
Yes	1.08 (1.01, 1.15)	0.02	
No	1.07 (1.05, 1.09)	0.01	
DM			0.31
Yes	1.08 (0.90, 1.29)	0.41	
No	0.96 (0.85, 1.09)	0.56	
CKD			0.72
Yes	0.97 (0.83, 1.14)	0.73	
No	1.02 (0.86, 1.20)	0.86	
Hypertension			0.09
Yes	1.00 (0.87, 1.14)	0.98	
No	1.21 (1.00, 1.47)	0.05	
Stroke			0.58
Yes	0.97 (0.78, 1.19)	0.75	
No	1.03 (0.93, 1.14)	0.62	
CVD			0.18
Yes	0.90 (0.70, 1.14)	0.37	
No	1.08 (0.95, 1.21)	0.23	

## Data Availability

The data that support the findings of this study are publicly available in the National Health and Nutrition Examination Survey at https://wwwn.cdc.gov/nchs/nhanes.
